# Immune-Inflammatory Responses of an Acellular Cartilage Matrix Biomimetic Scaffold in a Xenotransplantation Goat Model for Cartilage Tissue Engineering

**DOI:** 10.3389/fbioe.2021.667161

**Published:** 2021-06-02

**Authors:** Litao Jia, Peiling Zhang, Zheng Ci, Wei Zhang, Yu Liu, Haiyue Jiang, Guangdong Zhou

**Affiliations:** ^1^Research Institute of Plastic Surgery, Weifang Medical University, Weifang, China; ^2^Research Center of Plastic Surgery Hospital, Chinese Academy of Medical Sciences and Peking Union Medical College, Beijing, China; ^3^Department of Plastic and Reconstructive Surgery, Shanghai Key Laboratory of Tissue Engineering, Shanghai Ninth People’s Hospital, Shanghai Jiao Tong University School of Medicine, Shanghai, China; ^4^National Tissue Engineering Center of China, Shanghai, China

**Keywords:** immune-inflammatory responses, acellular cartilage matrix, biomimetic scaffold, xenotransplantation model, cartilage tissue engineering

## Abstract

The rapid development of tissue engineering and regenerative medicine has introduced a new strategy for ear reconstruction, successfully regenerating human-ear-shaped cartilage and achieving the first clinical breakthrough using a polyglycolic acid/polylactic acid (PGA/PLA) scaffold. However, its clinical repair varies greatly among individuals, and the quality of regenerated cartilage is unstable, which seriously limits further clinical application. Acellular cartilage matrix (ACM), with a cartilage-specific microenvironment, good biocompatibility, and potential to promote cell proliferation, has been used to regenerate homogeneous ear-shaped cartilage in immunocompromised nude mice. However, there is no evidence on whether ACM will regenerate homogeneous cartilage tissue in large animals or has the potential for clinical transformation. In this study, xenogeneic ACM assisted with gelatin (GT) with or without autologous chondrocytes was implanted subcutaneously into goats to establish a xenotransplantation model and compared with a PGA/PLA scaffold to evaluate the immune-inflammatory response and quality of regenerated cartilage. The results confirmed the superiority of the ACM/GT, which has the potential capacity to promote cell proliferation and cartilage formation. Although there is a slight immune-inflammatory response in large animals, it does not affect the quality of the regenerated cartilage and forms homogeneous and mature cartilage. The current study provides detailed insights into the immune-inflammatory response of the xenogeneic ACM/GT and also provides scientific evidence for future clinical application of ACM/GT in cartilage tissue engineering.

## Introduction

Auricular cartilage defects, including congenital auricle malformation–microtia and acquired auricular cartilage injuries caused by various causes are very common, and the most clinical effective treatment is reconstruction using autologous costal cartilage engraving, which can cause serious complications such as surgical trauma and pneumothorax (Brent, 2002; Zhang et al., 2009; Luquetti et al., 2011, 2012; Bly et al., 2016). Fortunately, the rapid development of tissue engineering and regenerative medicine technologies has introduced a new strategy for ear reconstruction, successfully regenerating human-ear-shaped cartilage and achieving the first clinical breakthrough based on a polyglycolic acid/polylactic acid (PGA/PLA) scaffold (Cao et al., 1997; Haisch, 2010; Zhou et al., 2018; Yin et al., 2020). However, the degradation of implanted polymer scaffolds can cause aseptic inflammation and lead to unstable cartilage regeneration, which seriously limits its further clinical application (Ceonzo et al., 2006; Luo et al., 2013; Liu et al., 2016). Therefore, an appropriate scaffold with good biocompatibility and low immunogenicity is required for cartilage regeneration. Recent studies have found that acellular cartilage matrix (ACM) has a cartilage-specific microenvironment, good biocompatibility, and the potential to promote cell proliferation (Xue et al., 2012; Li Y. et al., 2019; Wiggenhauser et al., 2019; Jian et al., 2021). Combined with 3D printing, cast molding, gelatin (GT) assisted crosslinking, and freeze-drying techniques, a human-ear-shaped scaffold based on ACM has been successfully prepared and used to regenerate homogeneous ear-shaped cartilage in immunocompromised nude mice (Jia et al., 2020). Nevertheless, it is still unproven whether ACM/GT scaffolds will trigger immune-inflammatory responses like PGA/PLA scaffolds and whether ACM/GT scaffolds will regenerate homogeneous cartilage tissue in the subcutaneous environment of large animal models.

To address the issues mentioned above, it is necessary to systematically compare the post-implantation reaction and cartilage regeneration of ACM/GT scaffolds and PGA/PLA scaffolds (which were previously applied clinical) in large animals with sound immune function to predict the possible advantages of ACM/GT scaffolds in reducing immune-inflammatory response and enhancing the quality of regenerated cartilage in future clinical translation. Therefore, the main purpose of this current study is to verify whether ACM/GT scaffold can induce serious immune-inflammatory responses in large animals with sound immune function and whether mature homogeneous cartilage can be successfully regenerated in the subcutaneous environment.

To effectively verify the immune-inflammatory response of xenogeneic ACM in large animals with sound immune function, ACM derived from cow scapular cartilage assisted with gelatin was implanted subcutaneously in goats to establish the xenotransplantation model used in this study. A PGA/PLA scaffold was also implanted subcutaneously to compare immune-inflammatory responses. Two groups of scaffold constructs inoculated with autologous auricular chondrocytes were also implanted subcutaneously in goats to explore the quality of cartilage regeneration and long-term stability *in vivo*. The current study provides detailed insights into the immune-inflammatory responses and cartilage regeneration stability of ACM/GT in the xenotransplantation model and scientific evidence for future clinical application in cartilage tissue engineering.

## Materials and Methods

### Preparation of the Scaffolds

Cartilage pieces obtained from cow scapular cartilage were processed into ACM powder after freeze–grinding and decellularization procedures. The ACM scaffold, using gelatin extracted from bovine Achilles tendon (Gel strength approximately 240 g Bloom, Aladdin) as an auxiliary crosslinker, and PGA/PLA scaffold were fabricated according to our previously reported protocols (Liu et al., 2010; Jia et al., 2020). Briefly, the ACM particles were filtered by 100-mesh (150 ± 10 μm) filtration screens to obtain uniform particles. A certain concentration of gelatin solution (dissolved in deionized water) was first placed at 4°C as a gel for an hour to reduce ACM particles deposition as much as possible before fully mix. Then the suspension was fully frozen at −10°C for 24 h. The specific concentration and proportion of the ACM suspension and GT solution (2% concentrations, ACM: GT = 5:5) optimized in our previous experiment were blended uniformly and freeze-dried to form a porous ACM/GT scaffold. Thirty micrograms of unwoven PGA fibers (National Tissue Engineering Center of China, Shanghai, China) were compressed into cylindrical scaffolds (8 mm in diameter and 2 mm in thickness) and then 1.0% PLA (Sigma, St. Louis, MO, United States) solubilized in dichloromethane was continuously added to form the PGA/PLA scaffold. All scaffolds were sterilized before application using ethylene oxide.

### Scanning Electron Microscopy (SEM)

The surface morphology of the two kinds of scaffolds was observed by SEM (Philips XL-30, Amsterdam, Netherlands) at an accelerating voltage of 15 kV. The two kinds of cell–scaffold constructs cultured for 7 days *in vitro* were washed with PBS and fixed overnight in 0.05% glutaraldehyde at 4°C. After dehydration in a graded ethanol series and critical point drying, the surface morphology and extracellular matrix (ECM) production of these constructs were observed by SEM (Chen et al., 2016).

### Porosity Analysis of the Scaffolds

The porosity of the ACM/GT scaffold and PGA/PLA scaffold was tested using the ethanol infiltration method as previously described (Serra et al., 2015). Briefly, V1 and V2 were marked as the volume of ethanol in the measuring cylinder before and after the scaffold was immersed in ethanol, respectively. V3 was marked as the remaining ethanol volume after the scaffold was removed from ethanol. The porosity of the scaffold (*n* = 5 per group) was calculated using the following formula: Porosity = (V1–V3)/(V2–V3) × 100%.

### Biocompatibility of the Scaffolds

#### Cell Seeding Efficiency

Chondrocytes from the auricular tissue of goats were isolated and cultured in a basic medium that Dulbecco’s modified Eagle medium (DMEM, Gibco) containing 10% fetal bovine serum (FBS, Gibco) at 37°C with 95% humidity and 5% CO_2_ according to the previously established methods (Zhang et al., 2014). Harvested chondrocytes from the second passage were adjusted to a final concentration of 75 × 10^6^ cells/mL, and 200 μL cell suspension was inoculated into each scaffold (*n* = 5 per group). After 24 h of incubation, the remaining cells were collected, counted, and the cell seeding efficiencies of the two groups were calculated based on the formula: (total cell number-remaining cell number)/total cell number × 100% (Zheng et al., 2014).

#### Cellular Viability Assessment

After 1, 4, 7, and 14 days of culture in basic medium, the cellular viability of the seeded chondrocytes on the ACM/GT scaffold and PGA/PLA scaffold (*n* = 5 per group) was evaluated using the Live and Dead Cell Viability Assay (Invitrogen, Carlsbad, CA, United States) following the manufacturer’s instructions, and examined by confocal microscope (Nikon, Japan) (Xu et al., 2020a).

#### Cellular Proliferation Assessment

After 1, 7, and 14 days of culture in basic medium, the cellular proliferation capacity of chondrocytes on the ACM/GT scaffold and PGA/PLA scaffold (*n* = 5 per group) was assessed by the total DNA quantification assay (PicoGreen dsDNA assay, Invitrogen, Carlsbad, CA, United States) following the manufacturer’s protocols (Xia et al., 2018).

### *In vitro* Culture of the Cell–Scaffold Constructs

Chondrocytes were collected and seeded evenly into each scaffold to form cell–scaffold constructs according to a previously described method (Xue et al., 2013). The constructs were incubated for 4 h at 37°C to allow for complete adhesion of the cells to the scaffolds and then cell–constructs were cultured for 2 weeks in the basic medium at 37°C with 95% humidity and 5% CO_2_.

### Subcutaneous Implantation in Goats

Six 3-month-old goats (4 males and 2 females) were purchased from Shanghai Jiagan Biological Technology Co., Shanghai, China. All protocols with animal use were approved by the Animal Care and Experiment Committee of Shanghai Jiao Tong University School of Medicine (Shanghai, China). Six goats were divided into two groups: in three goats, two kinds of scaffolds without cells (PGA/PLA scaffolds and ACM/GT scaffolds, *n* = 10 scaffolds per group in each goat) were implanted subcutaneously into both sides of the abdomen of each goat. Five samples per group in each goat were harvested after 1 and 2 weeks of implantation for subsequent analysis, respectively. In the other three goats, two kinds of cell–scaffold constructs (cell-PGA/PLA constructs and cell-ACM/GT constructs, *n* = 15 constructs per group in each goat) cultured for 2 weeks *in vitro* were implanted subcutaneously into both sides of the abdomen of each donor goat for continue culture *in vivo*. Five samples per group in each goat were harvested after 1, 2, and 8 weeks of implantation for subsequent analysis, respectively. During surgery, each goat was anesthetized and subcutaneous pockets were created in the abdominal area in which the scaffolds and constructs were implanted. After closure of the incisions, the animals were allowed to recover from the anesthesia.

### Immune-Inflammatory Response Evaluations

After 1 and 2 weeks of implantation, two kinds of constructs and two kinds of scaffolds (*n* = 5 samples per group in each goat) were harvested with surrounding tissue for analysis of the immune-inflammatory response. After gross observation, all samples were fixed in 4% paraformaldehyde for 48 h, embedded in paraffin, and sectioned in 5-mm slices according to our previously established methods (Liu et al., 2016). The slices were stained with hematoxylin and eosin (H&E), as well as immunohistochemical techniques as previously reported. CD3 was detected using rabbit anti-CD3 monoclonal antibody (ab16669, 1:200, Abcam, Cambridge, United Kingdom), followed by goat anti-rabbit IgG H&L (HRP) (ab205718, 1:2000, Abcam). CD 68 was detected using mouse anti-CD68 monoclonal antibody (ab955, 1:200, Abcam), followed by goat anti-mouse IgG H&L (HRP) (ab205719, 1:2000, Abcam). The quantification of CD3 and CD68 position area (%) was performed with Image J and IHC Profiler Software (*n* = 5 per group).

### Histological and Immunohistochemical Evaluations of Regenerative Tissues

After 8 weeks of culture *in vivo*, the two kinds of tissue-engineering cartilage tissue (*n* = 5 samples per group in each goat) were carefully harvested. Part of each sample (the rest of the sample was used for subsequent biochemical and biomechanical analysis) was prepared for histological and immunohistochemical analyses after gross observation and measurement, as described above (Li D. et al., 2019). These slices were stained with H&E, Safranin O, and type II collagen (COL II) to evaluate the histological structure and cartilage ECM deposition of tissue-engineering cartilage tissue. COL II was detected using rabbit anti-collagen II polyclonal antibody (ab34712, 1:100, Abcam), followed by goat anti-rabbit IgG H&L (HRP) (ab205718, 1:2000, Abcam). The quantification of regenerated cartilage area (%) was performed with Image J and IHC Profiler Software (*n* = 5 per group).

### Biochemical and Biomechanical Analysis

Two groups of tissue-engineering cartilage tissue cultured for 8 weeks *in vivo* were collected, weighed with an electronic balance, and the volume of each sample was measured by the water displacement method (*n* = 5 per group). Biochemical and biomechanical analysis of both regenerated tissues and native auricular cartilage was performed as described previously (Xu et al., 2020b). All samples were collected and minced to conduct cartilage-related biochemical evaluations (*n* = 3 per group). Briefly, glycosaminoglycan (GAG), total collagen, and DNA quantifications were quantified by the dimethyl methylene blue assay (DMMB, Sigma-Aldrich, St. Louis, MI, United States), hydroxyproline assay kit (Sigma-Aldrich), and PicoGreen dsDNA assay (Invitrogen, Carlsbad, CA, United States), respectively. The biomechanical analysis was determined using a mechanical testing machine (Instron-5542, Canton, MA, United States). All samples (*n* = 5 per group) were processed into a cylindrical shape and a constant compressive strain rate of 0.5 mm/min was applied until 80% of the maximal deformation. The Young’s modulus of each sample was calculated based on the slope of the stress–strain curve in the range of 0 to 40%.

### Statistical Analysis

All quantitative data were collected from at least three replicate tests, and values were expressed as mean ± standard deviation. After confirmation of the normal data distribution, a one-way analysis of the variance was used to determine the statistical significance among groups using GraphPad Prism 8 software, and a value of *p* < 0.05 was considered statistically significant.

## Results

### Characterization of the Scaffolds

**FIGURE 1:**
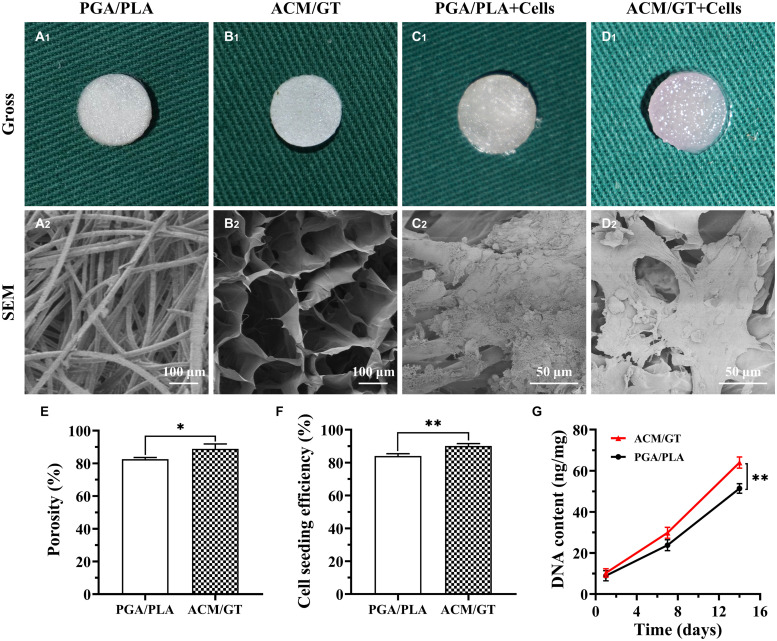
Characterization and biocompatibility of the scaffolds. Gross and SEM images of PGA/PLA scaffold **(A)**, ACM/GT scaffold **(B)**, as well as cell-scaffold constructs **(C,D)**. (**C_1_,D_1__)_** Were the immediate gross images of chondrocytes seeded on scaffolds, while **(C_2_,D_2__)_** were the SEM images of cell-scaffold constructs after 7 days of culture *in vitro*. Characterization analysis of the porosity of two scaffolds **(E)**. Cell seeding efficiency **(F)** and DNA content **(G)** of chondrocytes inoculated into the PGA/PLA scaffold and ACM/GT scaffold. Statistical significance: ^∗^*p* < 0.05, ^∗∗^*p* < 0.01.

Unwoven PGA fibers coated by PLA were compressed to form a cylindrical scaffold for imaging. Both scaffolds exhibited a porous structure and the SEM examination further confirmed the porous surface (Figures 1A,B). The porosity of the ACM/GT scaffold was greater than the PGA/PLA scaffold (Figure 1E). Overall, the two scaffolds met the requirements of cartilage tissue regeneration; however, the ACM/GT scaffold was more suitable because of the greater porosity for cell inoculation.

### Biocompatibility of the Scaffolds

**FIGURE 2:**
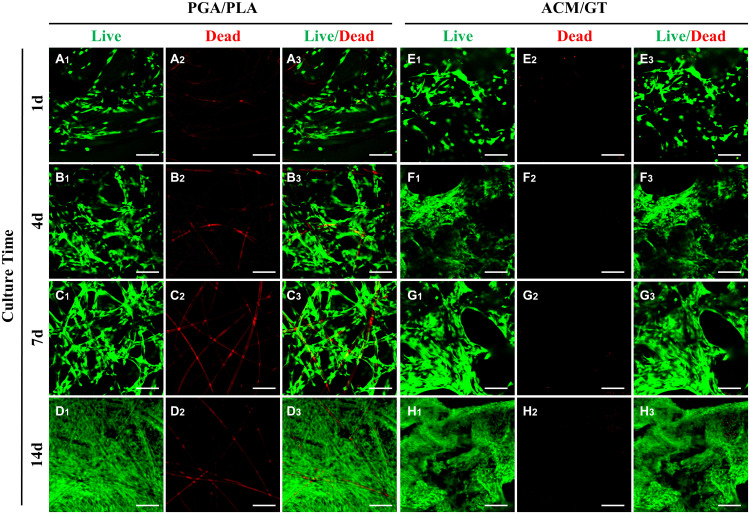
Evaluation of cell viability on the scaffolds. Live/Dead staining showed the number of chondrocytes gradually increased on the PGA/PLA scaffold **(A–D)** and ACM/GT scaffold **(E–H)** as the culture time increased *in vitro*. Almost all chondrocytes survive well (green cells) and few dead cells (red cells) are observed at all observation points. The linear red color (**A_2_–D_2_**, non-specific staining) is the PGA fibers. Scale bar: 100 μm.

After analyzing the characterization of the scaffolds, the cell biocompatibility was the focus of the next evaluation for cartilage tissue regeneration. Cell seeding efficiency, SEM, DNA content, and cellular viability analyses were performed to evaluate the biocompatibility of the two scaffolds. After cell seeding, the two scaffolds maintained their original shape and size, and gross observation showed that the cell suspension was quickly absorbed by the whole scaffolds (Figures 1C_1_,D_1_). After 7 days of culture *in vitro*, SEM observation revealed that the chondrocytes were well attached to the two scaffolds with a small amount of ECM production (Figures 1C_2_,D_2_). The cell seeding efficiency in the ACM/GT scaffold was more than 90%, which was significantly greater than on the PGA/PLA scaffold (Figure 1F). Cellular viability assays showed that chondrocytes grew well on the two scaffolds with significant proliferation over time, and very few dead cells were observed at all observation times (Figure 2). DNA quantitative analysis further indicated that the number of chondrocytes gradually increased with time on the two scaffolds. However, the DNA content of chondrocytes on the ACM/GT scaffold was significantly greater than on the PGA/PLA scaffold (Figure 1G), which indicates that ACM may have the potential to promote cell proliferation. Collectively, these *in vitro* results indicated that the ACM/GT scaffold was more favorable for cell adhesion and cell proliferation, indicating better biocompatibility of the ACM/GT scaffold when compared with PGA/PLA scaffold.

### Immune-Inflammatory Response Evaluations of the Scaffolds

**FIGURE 3:**
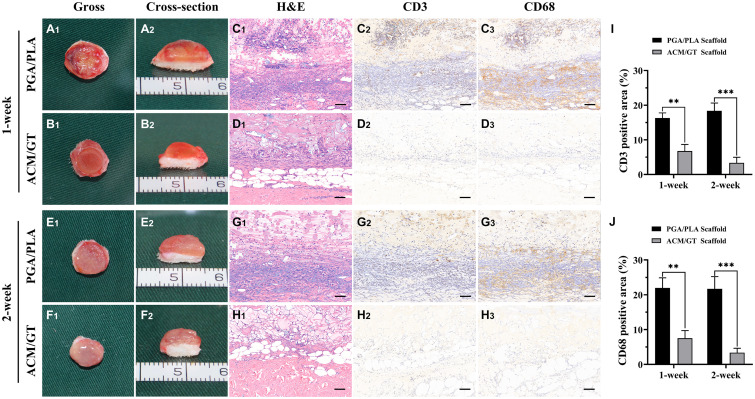
The immune-inflammatory response evaluations of the scaffolds subcutaneously implanted for 1 and 2 weeks. Gross and cross-section observation of the PGA/PLA scaffold and ACM/GT scaffold which subcutaneously implanted for 1 week **(A,B)** and 2 weeks **(E,F)**. Two groups of implanted samples with surrounding tissue were stained with H&E, CD3, and CD68 for immune-inflammatory response analysis **(C,D,G,H)** and the quantification of the CD3 **(I)** and CD68 **(J)** position area (%). Scale bar: 100 μm. Statistical significance: ^∗∗^*p* < 0.01, ^∗∗∗^*p* < 0.001.

To capture the early stage post-implantation inflammatory reaction, the two kinds of scaffolds were evaluated by H&E staining and immunohistochemical staining of lymphocytes (CD3) and macrophages (CD68) after 1 and 2 weeks of subcutaneous implantation. As shown in Figure 3, the contours of the two groups of implants were observed after 1 week of subcutaneous implantation. A large amount of cell infiltration was observed around the PGA/PLA scaffold while much milder infiltration was observed in the ACM/GT scaffold. The immunohistochemical staining results showed homogenous and strong positive staining of CD3 and CD68 around the PGA/PLA scaffold. After 2 weeks of implantation, the cell infiltration, CD3 and CD68 staining, as well as the quantification of CD3 and CD68 position area (%) around the ACM/GT scaffold were significantly reduced, while the degree of cell infiltration around the PGA/PLA scaffold was significantly increased, which may be because of an aseptic inflammatory reaction caused by the acidic degradation products of the PGA/PLA scaffold.

### Immune-Inflammatory Response Evaluations of the Cell–Scaffold Constructs

**FIGURE 4:**
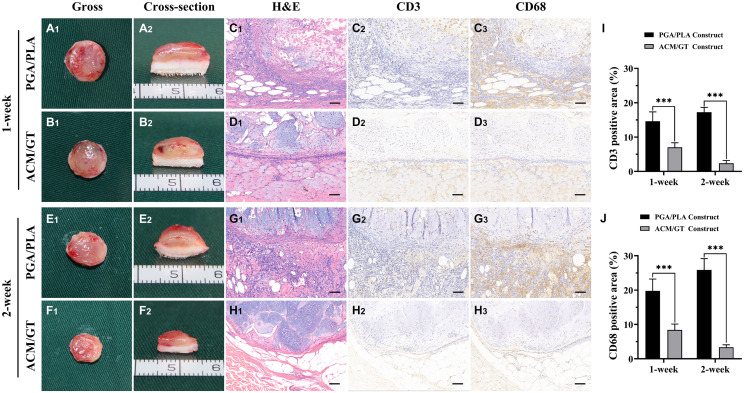
The immune-inflammatory response evaluations of the cell-scaffold constructs subcutaneously implanted for 1 and 2 weeks. Gross and cross-section observation of the cell-PGA/PLA construct and cell-ACM/GT construct which subcutaneously implanted for 1 week **(A,B)** and 2 weeks **(E,F)**. Two groups of implanted samples with surrounding tissue were stained with H&E, CD3, and CD68 for immune-inflammatory response analysis **(C,D,G,H)** and the quantification of the CD3 **(I)** and CD68 **(J)** position area (%). Scale bar: 100 μm. Statistical significance: ^∗∗∗^*p* < 0.001.

As shown in Figure 4, a large number of inflammatory cells adhered to the cell–scaffold constructs after 1 week of subcutaneous implantation. The boundary area of the ACM/GT group was clear and immature cartilage tissue, while the area of the PGA/PLA group was infiltrated and enveloped by inflammatory cells. Positive staining of CD3 and CD68 was also observed around the PGA/PLA constructs. After 2 weeks of subcutaneous implantation, the cell infiltration was significantly reduced at the outer edge of the ACM/GT construct and the cartilaginous islands were surrounded by a thin layer of negative staining of CD3 and CD68. The quantification of the CD3 and CD68 position area (%) confirmed the above histological results. Notably, the degree of cell infiltration around the PGA/PLA construct was remarkably increased and no typical cartilaginous island was observed compared with the ACM/GT scaffold. These results indicate that the PGA/PLA scaffold had a more severe inflammatory response to the host organism than the ACM/GT scaffold, potentially because chondrocytes promote the degradation of the PGA/PLA scaffold, triggering a serious aseptic inflammatory response and seriously affecting the formation of cartilage.

### Histological and Immunohistochemical Evaluations of Regenerative Tissues

**FIGURE 5:**
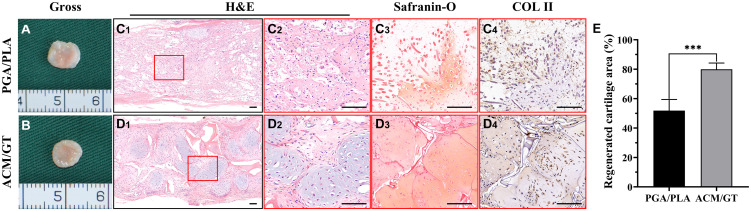
Gross view and histological examination of regenerated cartilage. Gross observation of the cartilage-like tissue regenerated by the cell-PGA/PLA construct **(A)** and cell-ACM/GT construct **(B)** which subcutaneously implanted for 8 weeks. Two groups of regenerated cartilage-like tissue were stained with H&E, Safranin-O, and COL II for the assessment of the quality of cartilage formation **(C,D)** and the quantification of the regenerated cartilage area (%) **(E)**. Scale bar: 100 μm. Statistical significance: ^∗∗∗^*p* < 0.001.

After 8 weeks of subcutaneous implantation into autologous goats, the two groups of regenerative tissue were harvested to explore the corresponding status of neo-cartilage formation *in vivo*. In the gross observation, the regenerative tissue formed by the PGA/PLA construct had a reddish appearance and soft texture (Figure 5A). However, the regenerative tissue formed by the ACM/GT construct also showed a reddish appearance, but with slippery and firmer texture (Figure 5B). Histological examinations further confirmed the results from gross observation, where the regenerative tissue formed by the PGA/PLA construct showed a fibrous tissue structure with abundant scaffold fibers, and no obvious cartilaginous tissue was detected (Figure 5C). The regenerative tissue formed by the ACM/GT construct showed typical cartilage features with abundant lacuna structures as well as positive staining of Safranin-O and collagen II, although the neo-cartilage tissue was not obviously homogenous (Figure 5D). The regenerated cartilage area (%) formed by the ACM/GT constructs were significantly greater than those formed by the PGA/PLA constructs, which supported the above histological results (Figure 5E). These results indicated that the ACM might have the potential to enhance the quality of cartilage regeneration in a subcutaneous environment.

### Biochemical and Biomechanical Analysis

**FIGURE 6:**
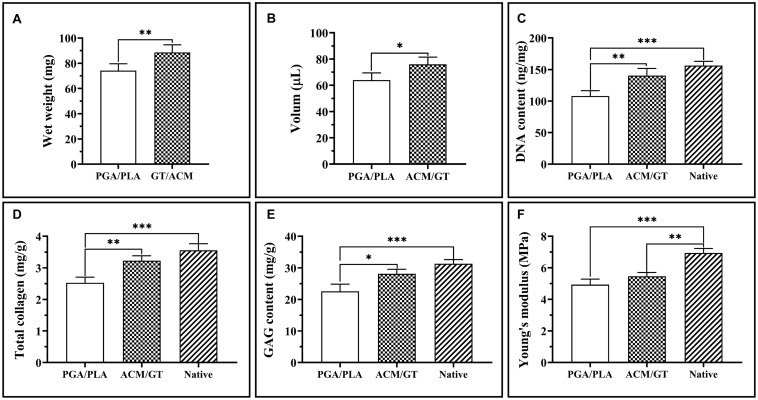
Quantitative analysis of regenerated cartilage. The wet weight and volume of the regenerated cartilage-like tissues by the cell-PGA/PLA construct and cell-ACM/GT construct which subcutaneously implanted for 8 weeks **(A,B)**. The DNA content **(C)**, total collagen **(D)**, GAG content **(E)**, and Young’s modulus **(F)** of cartilage-like tissue formatted by two cell-scaffold constructs. Statistical significance: ^∗^*p* < 0.05, ^∗∗^*p* < 0.01, ^∗∗∗^*p* < 0.001.

Naturally, the evaluation of final cartilage formation is the most important criterion to determine whether a scaffold can be suitable for cartilage tissue engineering. The quantitative analysis related to the neo-cartilage formation that is based on the regenerated tissue subcutaneously implanted for 8 weeks further supports the above results. The wet weight and volume of the neo-cartilage tissue formed by the ACM/GT constructs were significantly greater than those formed by the PGA/PLA constructs (Figures 6A,B). Similarly, the DNA content, total collagen, and GAG content in the ACM/GT group were close to native cartilage, though significantly greater than the PGA/PLA group (Figures 6C–E). In addition, Young’s modulus in the two groups was not statistically different, both reaching more than 75% of the native cartilage (Figure 6F). Quantitative analysis results showed that the quality of cartilage regeneration in the ACM/GT group was significantly better than that in the PGA/PLA group, indicating the ACM/GT scaffold was more suitable for cartilage tissue engineering than the PGA/PLA scaffold.

## Discussion

The rapid development of tissue engineering and regenerative medicine technology has provided new strategies for auricular reconstruction (Cervantes et al., 2013; Kang et al., 2016; Schroeder and Lloyd, 2017; Wiggenhauser et al., 2017; Jang et al., 2020). Morphological cartilage of human auricular tissue has been successfully regenerated using a PGA/PLA scaffold, and the first clinical breakthrough was achieved (Cao et al., 1997; Zhou et al., 2018). However, its clinical repair varies greatly among individuals, and the quality of regenerated cartilage is unstable, which seriously limits its further clinical application. The poor quality of regenerated cartilage may be related to the acidity of the degradation products of polymer materials, which easily causes an aseptic inflammatory reaction (Asawa et al., 2012; Kanazawa et al., 2013). After PGA/PLA scaffold implantation, acid degradation products accumulate in local tissue, and a large number of inflammatory cells gather and phagocytize the material fibers (Lu et al., 2000; Boland et al., 2004; Pamula and Menaszek, 2008). Concurrently, a series of chemical factors are released, which cause a strong inflammatory response of the host and affect the adhesion of chondrocytes and matrix secretion. The fibrous connective tissue stimulated by inflammation is mixed into the constructed tissue, which weakens the construction quality of the tissue engineered cartilage (Grizzi et al., 1995; Li and McCarthy, 1999). Overall, the engineered cartilage is easily damaged by a severe immune reaction in large animals, causing the implant to not form cartilage tissue well, which limits its transformation to clinical application. However, there are still some problems that need to be addressed for the subcutaneous model of large animals.

Recent studies have found that ACM has a cartilage-specific microenvironment, good biocompatibility, and the potential to promote cell proliferation (Yang et al., 2008, 2010; Utomo et al., 2015; Kim et al., 2020). In the early stage, with the integration of 3D printing, casting molding, and freeze-drying techniques, the human-ear-shaped scaffolds based on ACM have been successfully prepared and further inoculated with chondrocytes to regenerate homogeneous ear-shaped cartilage in immunocompromised nude mice (Jia et al., 2020). However, there is a great difference in the outcome of cartilage regeneration between small animal models and large animal models. That is, the results in small animal models are difficult to be replicated in large animal models, especially in immunocompromised nude mice, which cannot predict the feasibility of clinical application in the future. Therefore, it is necessary to verify the validity and predict the feasibility of clinical translation in pre-clinical large animal models. But so far, there is no specific report on the immune-inflammatory response and cartilage regeneration of ACM in large animal models. So, we specifically conducted current research to systematically compare the post-implantation response and cartilage regeneration of ACM/GT scaffolds and PGA/PLA scaffolds (which were previously applied clinical) in large animal models to predict the potential advantages of ACM/GT scaffolds in reducing immune-inflammatory response and enhancing the quality of regenerated cartilage.

In this study, the cell biocompatibility of ACM derived from cow scapular cartilage was first verified using *in vitro* experiments. The results showed the superiority of the ACM/GT scaffold compared with the PGA/PLA scaffold, especially in cell proliferation, which indicates that ACM may release soluble growth factors. And our previous studies have identified the presence of some growth factors, such as transforming growth factor-beta (TGF-β), insulin-like growth factor (IGF), and bone morphogenetic protein-2 (BMP-2), which have the potential to promote chondrocyte proliferation (Xue et al., 2012, 2018). This could provide a reasonable explanation for the superior biocompatibility of the ACM/GT scaffold.

To clarify the immune-inflammatory response, xenogeneic ACM/GT with or without autologous chondrocytes was implanted subcutaneously into goats to establish the xenotransplantation model. There was cell infiltration of ACM/GT at an early stage, which was significantly alleviated by 2 weeks, indicating a mild inflammatory response to the host organism. However, the PGA/PLA scaffold showed cell infiltration, which worsened significantly by 2 weeks. This may be induced by the acidic products that are gradually degraded from the PGA/PLA scaffold (Liu et al., 2016). Notably, the immune-inflammatory response of the cell–PGA/PLA scaffolds was more severe, potentially because the chondrocytes promoted degradation of the PGA/PLA scaffold and triggered a severe aseptic inflammatory response.

Furthermore, testing the quality and long-term outcome of tissue-engineered cartilage formatted *in vivo* is the necessary step of preclinical verification. The evaluation of tissue-engineered cartilage showed the ACM/GT construct formed a typical, mature, and homogeneous cartilage tissue, indicating the mild inflammation does not affect cartilage regeneration and the ACM may have the potential to promote the formation of cartilage tissue. Alternatively, no typical cartilage features were observed in the regenerative tissue by the PGA/PLA scaffolds, potentially because of the aseptic inflammatory response hindering the formation of cartilage.

The current research confirmed the potential of ACM to promote cell proliferation and cartilage formation *in vivo* and i*n vitro* experiments. Although there is a slight immune-inflammatory response in large animals that have a sound immune system, it does not affect the quality of the regenerated cartilage. The mild immune-inflammatory response may be related to the remaining galactosyl-alpha-(1,3)-galactose (α-Gal), which is immunogenic in most mammalian species including humans and causes glycan-specific IgG and IgE responses with clinical relevance (Jappe et al., 2018; Bernth Jensen et al., 2020). In the next study, α-Gal in ACM will be removed to obtain a lower immunogenic scaffold. The homogeneous and mature cartilage can be regenerated and maintain long-term stability, which suggests that ACM/GT can replace the PGA/PLA scaffold to improve clinical repair. Additionally, as a natural biodegradable material, ACM has more advantages in microenvironment bionics, biocompatibility, and biosafety, for more prospects of future clinical transformation.

## Conclusion

In summary, the current study confirmed the superiority of the ACM to PGA/PLA scaffolds with the potential to promote cell proliferation and cartilage formation. Although there is a slight immune-inflammatory response in large animals that have a sound immune system, it does not affect the quality of regenerated cartilage and can form homogeneous and mature cartilage. Although further research is needed to obtain the ACM with lower immunogenicity, the current results provide scientific evidence for its future clinical application in cartilage tissue engineering.

## Data Availability Statement

The original contributions presented in the study are included in the manuscript/supplementary material, further inquiries can be directed to the corresponding authors.

## Ethics Statement

The animal study was reviewed and approved by the Animal Care and Experiment Committee of Shanghai Jiao Tong University School of Medicine (Shanghai, China).

## Author Contributions

LJ: conceptualization, methodology, data curation, formal analysis, and writing original draft. PZ: conceptualization, data curation, and software. ZC and WZ: conceptualization and methodology. YL: conceptualization and supervision. HJ: conceptualization, methodology, and supervision. GZ: conceptualization, methodology, and writing review and editing. All authors contributed to the article and approved the submitted version.

## Conflict of Interest

The authors declare that the research was conducted in the absence of any commercial or financial relationships that could be construed as a potential conflict of interest.
